# Fabrication and Analysis of Chemically-Derived Graphene/Pyramidal Si Heterojunction Solar Cells

**DOI:** 10.1038/srep46478

**Published:** 2017-04-11

**Authors:** Wen-Chieh Lee, Meng-Lin Tsai, You-Ling Chen, Wei-Chen Tu

**Affiliations:** 1 Department of Electronic Engineering, Chung Yuan Christian University, Taoyuan, 320, Taiwan (R.O.C.); 2 Department of Materials Science and Engineering, National Tsing Hua University, Hsinchu, 300, Taiwan (R.O.C.)

## Abstract

In the study, the chemically-derived reduced graphene oxide flakes on the pyramidal Si substrate to construct the heterojunction solar cells *via* simple spin-coating process have been presented. The total reflectance of chemically-derived graphene on pyramidal Si is ~12% at the wavelength of 550 nm which is remarkably reduced compared with that of reduced graphene oxide on planar Si. By modifying the density and distribution of reduced graphene oxide flakes on Si, the power conversion efficiency of 5.20% is achieved. Additionally, the simulated absorbance of different-thick graphene is implemented to optimize the performance of graphene/pyramidal Si devices. The fabrication technique for rGO-based devices has the merits of simplicity, large scale, high throughput and low cost, which is a new starting point in the direction of graphene-based material for the applications of next generation optoelectronics.

Developing cost-effective devices for energy storage and conversion are the major and urgent issue to overcome the global warming and climate change. Consequently, seeking a low-cost and high-efficient alternative structure in the form of heterojunction solar cells is in accordance with the roadmap of solar cells^1^,^2^. A graphene film with attractive properties such as high conductivity, high transparency, flexible, and wide-band absorption^3^,^4^, has been proposed for next-generation optoelectronic materials and devices in the applications ranging from solar cells, light emitting diodes, photodetectors to biosensors^5^,^6^,^7^,^8^. In particular, graphene incorporated with planar Si wafer forms a Schottky contact and introduces a built-in electric field at the graphene/Si interface, which may potentially be used for heterojunction solar cells. Li *et al*. proposed one of the earliest study in constructing graphene-based solar cells by transferring graphene sheets on n-type Si surface which exhibited a PCE of 1.5%^9^. It was also noticed that the performance of graphene/Si solar cell could be optimized by controlling the sheet resistance and the doping of graphene film^10^,^11^. Subsequently, much effort has been made to improve the PCE of graphene/Si solar cells by doping or treating graphene films. For example, a power conversion efficiency (PCE) of ~6.1% was achieved by HNO_3_ treated graphene woven fabrics leading to the increased work function, the carrier density, and the built-in potential. Additionally, a PCE of ~8.6% was performed by using bis(trifluoromethanesulfonyl)-amide[((CF_3_SO_2_)2NH)] (TFSA) doped graphene. Unfortunately, the graphene films are typically grown by chemical vapor deposition (CVD) under high temperature and high vacuum condition^12^. Moreover, the sizes of these graphene-based solar cells are usually limited in micrometer scale that impedes the development of graphene-based solar cells on an industrial scale for the future energy value chain. Consequently, chemically-derived reduced graphene oxide (rGO) is a promising solution to the issue and thus the construction of cost-effective rGO/Si solar cells will be beneficial for next-generation industrial production^13^. However, the efficiency of rGO/Si solar cells cannot compete with graphene/Si solar cells till now.

To improve the performance of rGO/Si solar cells, the formation of structured Si is one of the promising key to enhance the efficiency because the reflection of Si would be reduced and then the light path length can be increased. In this work, the chemically-derived rGO flakes spin-coated on planar and pyramidal Si substrates to construct rGO/Si heterojunction solar cells are realized. Morphologies of the planar and pyramidal Si solar cells with rGO flakes are observed by scanning electron microscopy (SEM) and atomic force microscopy (AFM). To ensure the rGO is reduced from graphene oxide (GO) and the resistivity of rGO on textured Si is similar to that of rGO on planar Si and, the sheet resistance (R_S_) of GO/planar Si, GO/pyramidal Si, rGO/planar Si and rGO/pyramidal Si is measured by Hall effect measurement system. To confirm the quality of rGO, the Raman spectra are carried out with a laser excitation of 514 nm. The Fourier transform infrared spectroscopy (FTIR) measurement of GO and rGO are also taken to demonstrate the oxygen related groups derived from the intensive oxidation are eliminated. The reflectance and absorbance spectra of planar and pyramidal Si with rGO are characterized to prove the antireflection effect of the textured structure. The parameters of solar cells are measured by a Keithley 2400 source meter with an air mass (AM) 1.5 filter to generate simulated AM 1.5 G illumination. By optimizing the uniformity of rGO flakes on pyramidal Si, the PCE of 5.20% is achieved which shows a significant improvement compared with the rGO/planar Si solar cell of 2.96% in PCE. Additionally, the absorbance spectra of different-thickness graphene on pyramidal Si and the electric field intensity distribution are simulated by finite-differentiate time-domain (FDTD) method. By controlling the effective thickness of graphene flakes, the absorbance is modified, leading to an optimized performance of graphene-based devices. This research provides a technique for the fabrication of large-scale rGO films on textured silicon *via* cost-effective spin-coating process which gives an insight for the future applications of rGO optoelectronic devices.

## Methods

**Figure 1:**
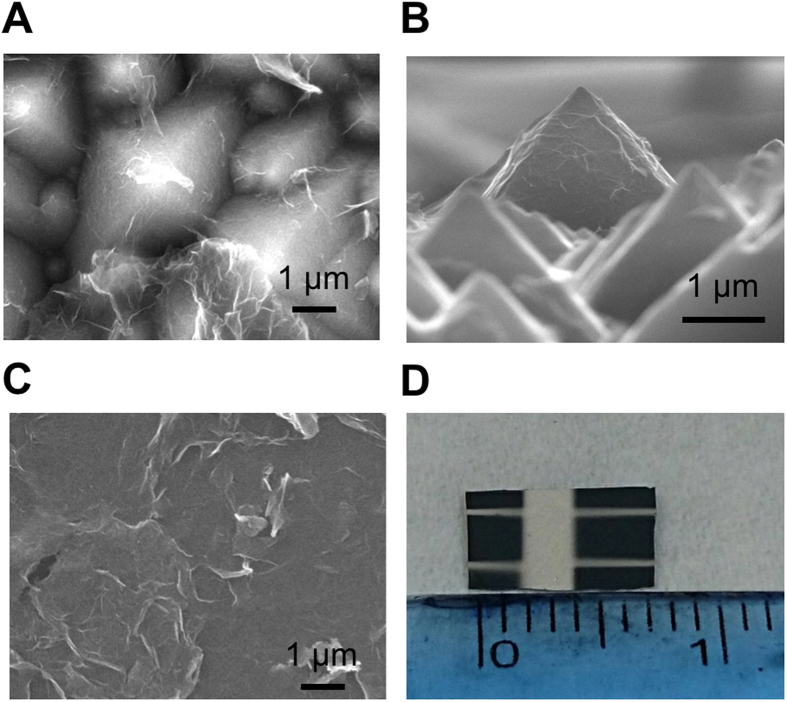
SEM and photographic images of rGO/Si solar cells. (**A**) Top-view and (**B**) Cross-sectional view SEM images of rGO/pyramidal Si solar cell. (**C**) SEM image of rGO on planar Si. (**D**) Photograph of the rGO/pyramidal Si heterojunction solar cell with top Ag electrode. The width and length of solar cell is 5 mm and 7 mm, respectively.

**Figure 2:**
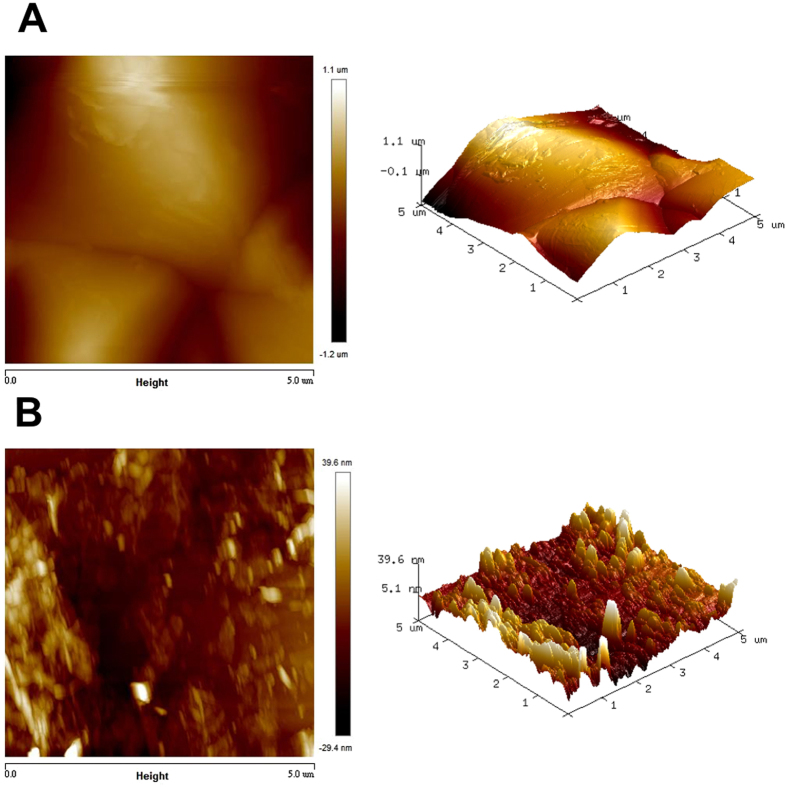
Surface morphology of rGO/Si. AFM images of rGO on (**A**) pyramidal and (**B**) planar Si substrate with the scan size is 5 μm. The morphology in (**A**) reveals a good distribution of graphene on pyramidal Si and some series corrugations resulted from the stack of the rGO flakes are observed in (**B**).

We deposited rGO on pyramidal n-Si wafer *via* spin-coating process to construct rGO/Si heterojunction solar cells. First, the alkaline saw damage was removed by a potassium hydroxide (KOH) solution and then square-based pyramids on the crystalline Si wafer with a (100) orientation were formed due to anisotropic wet etching. The samples were then rinsed in deionized (DI) water, cleaned in hydrochloric acid (HCl) and hydrofluoric acid (HF) followed by rinsed in DI water. Prior to spin-coating graphene flakes on pyramidal Si, Al film and Ag bars served as bottom contact electrodes were deposited on Si. Subsequently, the native oxide film was removed using a diluted HF. The samples were then exposed to ambient air for oxygen passivation of dangling bonds to reduce surface states. To deposit graphene flakes suspension on top of solar cells, the chemically-derived GO and rGO was fabricated for mass production in the future. First, the GO was obtained by the modified Hummers’ methods^14^. Then 0.2 g GO in 200 ml DI water was sonicated in a flask followed by adding hydrobromic acid (6 ml, 40 wt.%) into the GO colloids. Subsequently, the mixtures were refluxed in an oil bath at 110 °C for 24 hours. After the filtration, washing and desiccation, rGO flakes were produced^15^,^16^. Then rGO flakes were spin coated on pyramidal Si with a spin rate of 2750 rpm and a 100 nm-thick Al/Ag and Ag served as back and top electrodes, respectively, were deposited on rGO/pyramidal Si surface by sputtering (Fig. 1A,B). Additionally, planar Si and pyramidal Si without coated graphene were prepared to distinguish the effect of graphene on performance of solar cells. It was revealed that several wrinkles at the edges of rGO flakes and multilayer of rGO flakes stack on the pyramidal Si with good adhesion. To compare the effect of surface structure on the performance of solar cells, rGO flakes on the planar Si was fabricated with the same parameters except the surface morphology of Si substrate (Fig. 1C). The sizes of large-area graphene/pyramidal Si solar cells are 7 mm in length and 5 mm in width (Fig. 1D). It is noted that upon proper spin rate of spin-coating process, the uniform rGO films are constructed, suggesting great performance of solar cells. To evaluate the uniformity and the size of rGO flakes, the 2D and 3D AFM of rGO on pyramidal and planar Si have been characterized (Fig. 2A,B). Based on the analysis of AFM images, the roughness of rGO flakes is around 10 ~ 20 nm and the lateral size is 1~3 μm.

## Results and Discussion

To demonstrate the rGO is reduced from GO and ensure the resistivity of rGO on different substrate is similar, the sheet resistance of GO/planar Si, GO/pyramidal Si, rGO/planar Si and rGO/pyramidal Si is measured. The sheet resistance of GO/planar Si and GO/pyramidal Si is around 4256 ~ 5534 Ω/square, while the sheet resistance of rGO/planar Si and rGO/pyramidal Si is significantly reduced to 88 ~ 300 Ω/square. The sheet resistance of GO either on planar or pyramidal Si substrate is expected to be higher than that of rGO^17^,^18^. This decrease in the sheet resistance verifies that the achievement of the reduction and ensure the conductivity of rGO on planar and textured substrate is similar.

**Figure 3:**
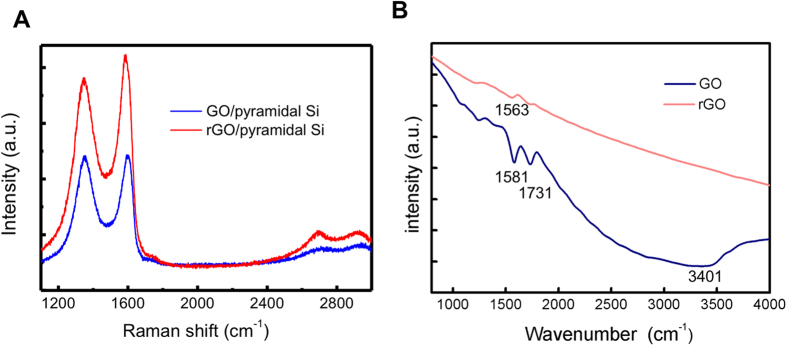
Optical characterizations of GO and rGO. (**A**) Raman spectrum of GO and rGO on pyramidal Si. (**B**) FTIR spectra of GO and rGO. The reduction of D peak in Raman spectrum and the elimination of oxygen related peak in FTIR demonstrate that the rGO is successfully derived.

The Raman spectrum of solution-processed GO and rGO flakes cast on the pyramidal Si with a 514 nm laser are measured to characterize the reduction degree of GO and the quality of rGO(Fig. 3A). The Raman spectrum of GO on pyramidal Si is characterized by D-band at 1352 cm^−1^, G band at 1591 cm^−1^ and almost no 2D-band, respectively. After the reduction, the Raman spectrum of rGO flakes is characterized by D-band at 1351 cm^−1^, G band at 1587 cm^−1^ and 2D-band at 2691 cm^−1^, respectively. The G band is ascribed to the Stokes Raman scattering with the E_2g_ phonon of sp^2^ C atoms and the presented D band is attributed from a breathing mode of ҡ-point photons of A_1g_ symmetry^19^,^20^. Compared with G peak of graphite (1580 cm^−1^), the G band of the rGO sample is up-shifted due to the presence of isolated double bonds that resonate at the higher frequencies. In addition, the G band at 1587 cm^−1^ is close to that of pure graphite^21^,^22^, revealing that the GO is reduced. Furthermore, the sharper 2D-band at 2691 cm^−1^ compared with 2D-band of GO also indicates the achievement of reduction. The decrease of D/G ratio after the reduction demonstrates that there are less oxygen related groups in rGO^23^,^24^. Additionally, the rGO flakes is obtained by reducing GO, therefore, the 2D/G ratio in Raman spectrum is lowered than that of single-layer graphene.

Figure 3B shows the typical FTIR spectra of GO and rGO. For the FTIR spectrum of GO, the vibration and deformation bands of O-H at 3401 cm^−1^, the stretching vibration band of C=O at 1731 cm^−1^, showing that there are abundant oxygen containing groups inserted into carbon skeleton. After the reduction, the intensities of the FTIR peaks related to the oxygen containing groups is reduced obviously and there is an appearance of a new peak at 1563 cm^−1^ owing to the skeletal vibration of graphene sheets, which reveals that these functional groups derived from the oxidation have been eliminated.

**Figure 4:**
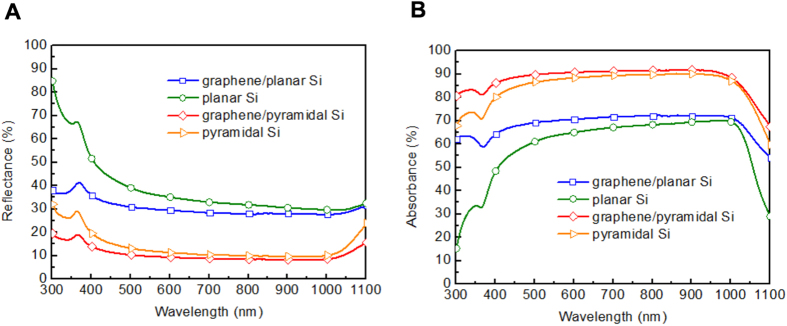
Optical characterizations of planar and pyramidal Si with and without graphene. (**A**) Reflectance and (**B**) absorbance spectra of graphene/planar Si, planar Si, graphene/pyramidal Si and pyramidal Si. Compared with graphene/planar Si, graphene on pyramidal Si displays the reduced reflectance and higher absorbance in the entire wavelength range owing to the effective antireflection.

To determine the antireflection effect of the textured Si, the reflectance (Fig. 4A) and absorbance (Fig. 4B) spectra of graphene/planar Si, planar Si, graphene/pyramidal Si and pyramidal Si were measured by spectrophotometer at the wavelength ranging from 300 nm to 1100 nm. The reflectance value of graphene on planar Si and planar Si at the wavelength of 550 nm is about 36% and 30%, respectively. By employing the etching strategy to form the pyramidal Si, the reflectance of graphene on pyramidal Si and pyramidal Si is obviously suppressed to 12% and 9% at the wavelength of 550 nm, respectively. These results indicate that a strong antireflection effect by the textured Si surface and the inhibition of the energy transfer loss from the incident light^25^,^26^. To deeply investigate the optical property of graphene on planar and pyramidal Si, the absorbance spectra were measured as illustrated in Fig. 4B. At the wavelength of 550 nm, the absorbance of graphene/planar Si, planar Si, graphene/pyramidal Si and pyramidal Si is 70%, 64%, 90%, 87%, respectively. The enhanced absorbance of graphene on pyramidal and planar Si compared with samples without graphene reveals that graphene was successfully coated on Si. Compared with graphene/planar Si, graphene/pyramidal Si shows higher absorbance due to effective antireflection. Although the nanoscale roughed Si surface leads to better antireflection effect than that of microscale pyramidal Si, the increased surface recombination poses a critical issue for constructing a good graphene/Si contact. It is expected that the Si surface morphology and the density of graphene suspension play an important role in the performance of solution-processed graphene/Si solar cells.

**Figure 5:**
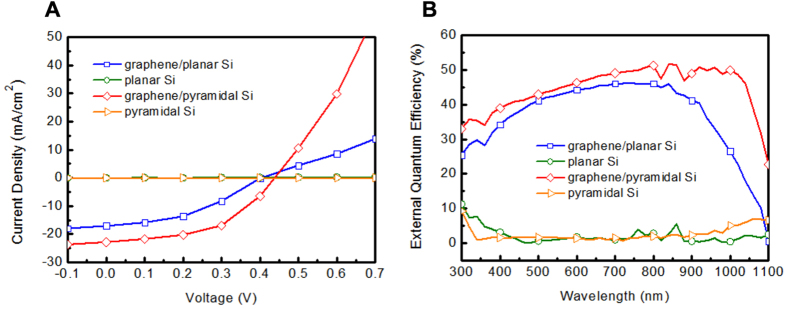
Photovoltaic characterizations of solar cells. (**A**) J–V curves and (**B**) EQE spectra of graphene/planar Si, planar Si, graphene/pyramidal Si and pyramidal Si structures.

The chemically-derived rGO with high optical bandgap is beneficial for costly heterojunction solar cells. The photovoltaic parameters of the rGO/pyramidal and rGO/planar Si solar cells are extracted by the measurement of current density-voltage (J-V) curves under AM 1.5G illumination. As the light incident on the sample, the generated carriers are separated by the built-in electric field at the graphene/Si interface and the holes are pulled to the graphene. As seen in Fig. 5A, planar Si and pyramidal Si without coated graphene do not exhibit solar cell characteristics because they have no effective Schottky junctions. The J-V characteristic of graphene/planar Si solar cell exhibits an open-circuit voltage (V_OC_) of 0.41 V, a J_SC_ of 18.26 mA/cm^2^ and a fill factor (FF) of 39.61%, which corresponds to a PCE of 2.96%. The parameters of V_OC_, J_SC_, FF and PCE for rGO/pyramidal Si are 0.44 V, 23.30 mA/cm^2^, 50.78% and 5.20%, respectively. The increase in V_OC_ and J_SC_ of rGO/pyramidal Si solar cell are resulted from the efficient antireflection effect and enhanced light path length, boosting the PCE significantly. In addition, this improvement can also be attributed to the lower series resistance and higher shunt resistance because of fewer wrinkle and stack of rGO on the surface of pyramidal Si compared with planar Si. Previous work on high-efficiency graphene/textured Si Schottky junction solar cells showed that the nanowire structure may induce severe recombination, leading to lower FF and PCF of the solar cells^27^. Compared with the nanowire structure, we propose the rGO/pyramidal structure which could be less rough and hence reduce the surface recombination.

To further examine the spectral response of rGO on pyramidal and planar Si substrates, the external quantum efficiency (EQE) of graphene on pyramidal and planar Si substrates are characterized (Fig. 5B). Planar Si and pyramidal Si without rGO show lower EQE spectra, while the EQE response of rGO/pyramidal Si solar cell over the entire excitation spectral range particularly in the long wavelength region is improved due to the increased light path length. The result indicates that graphene on the pyramidal Si surface might induce more photogenerated charge carriers due to pyramidal Si structure, leading to longer light path length and improved PCE of graphene-based solar cells.

**Figure 6:**
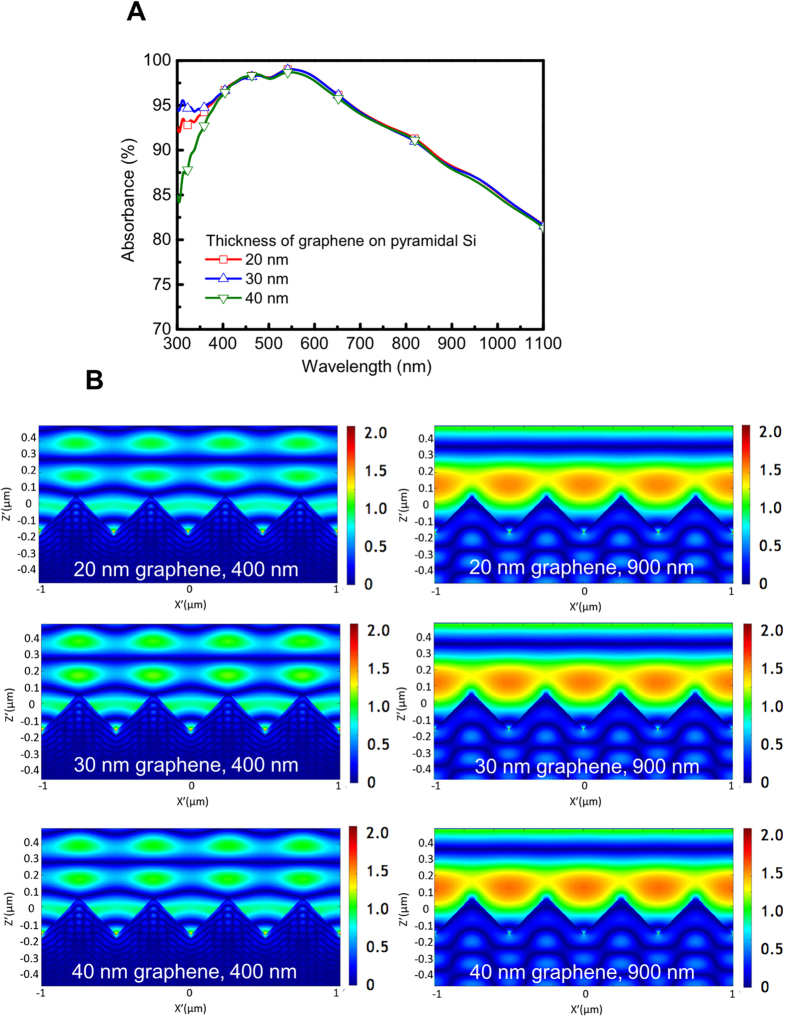
FDTD simulation. (**A**) Simulated absorbance spectra of different-thick graphene on pyramidal Si. The graphene film with 30 nm in thickness exhibits the highest absorption in the wavelength ranging from 300 nm to 400 nm. (**B**) The electric field intensity distribution of 30 nm, 40 nm, and 50 nm graphene on pyramidal Si at the wavelength of 400 nm and 900 nm.

For a better study of the mechanism of graphene heterojunction solar cells, a two-dimensional FDTD simulation of the graphene/pyramidal Si structure under illumination is performed to calculate the absorption spectra (Fig. 6A) and the electric field intensity distribution (Fig. 6B). The thickness of graphene layer is varied from 20 nm, 30 nm to 40 nm to investigate the thickness effect of graphene layer on the absorbance of graphene/Si structure. The simulated result predicts that the absorption values are highly sensitive to the thickness of graphene in the wavelength ranging from 300 nm to 400 nm. The 20 nm-thick and 30 nm-thick graphene on pyramidal Si shows the lowest and the highest absorption value, respectively; while the absorption value decreases with 40 nm-thick graphene because of some incident light is hard to penetrate graphene to Si. Consequently, the absorption of graphene flakes/pyramidal Si can be modified to obtain the high performance devices through the control of graphene layer. In Fig. 6B, the electric field intensity distributions for the 20 nm, 30 nm and 40 nm-thick graphene/pyramidal Si at the wavelength of 400 nm and 900 nm show that incident light is trapped inside the pyramidal structure, suggesting the efficiency of generating electron-hole pairs can be improved. At the wavelength of 400 nm, the electrical field intensity of 30 nm-thick graphene is highest among all samples while at the wavelength of 900 nm, the electrical field intensities of all samples are similar. These simulated electrical field intensities are in accordance with the absorbance spectra and superimposed on the performance of graphene/pyramidal Si solar cell compared with graphene/planar Si solar cell as shown in Fig. 5A.

## Conclusion

In conclusion, the field of graphene/Si heterojunction solar cells has rapidly developed to become the next-generation photovoltaics. To overcome the limited graphene size and the complicated transferring process, we propose a simple approach for constructing large-scale rGO on pyramidal Si through a cost-effective solution process. The pyramidal Si exhibits the superior antireflection effect, showing a substantial reduction in the reflectance of rGO/pyramidal Si compared with rGO on the planar Si substrate. Attributed to the uniform distribution of rGO on pyramidal Si, the performance of solar cell shows a promising PCE of 5.20%. Additionally, the simulated absorbance of different-thick graphene on pyramidal Si and the electric field intensity distribution are implanted with FDTD method to further optimize the characteristics of graphene/Si structure. The proposed approach and the analysis of solution-processed rGO flakes on large-scale and textured substrates have immense potential for graphene-based devices integrated into the future industrial production.

## Additional Information

**How to cite this article**: Lee, W.-C. *et al*. Fabrication and Analysis of Chemically-Derived Graphene/Pyramidal Si Heterojunction Solar Cells. *Sci. Rep.* **7**, 46478; doi: 10.1038/srep46478 (2017).

**Publisher's note:** Springer Nature remains neutral with regard to jurisdictional claims in published maps and institutional affiliations.
